# 

**Published:** 1935

**Authors:** 


### CONTENTS.

*Page*

The Twenty-fourth Long Fox Memorial Lecture: Observations on Pain. By MACDONALD CRITCHLEY, M.D., F.R.C.P. .. .. 191

Results of the Treatment of Mental Conditions. By G. DE M. RUDOLF, M.R.C.P., D.P.M., D.P.H. .. .. .. .. .. 219

Clinical Record: A Case of Pulmonary Consolidation of Doubtful Origin. By B. HUDSON, M.D., M.R.C.P., and F. L. WOLLASTON, M.D. .. .. .. .. .. .. .. 233

Reviews of Books .. .. .. .. .. .. .. .. 236

Editorial Notes .. .. .. .. .. .. .. .. 244

Obituaries. ROBERT ARTHUR ASKINS, M.A., M.D., D.P.H. .. .. 248

EDWIN VINCENT FOSS, M.B., Ch.B., M.R.C.S., L.R.C.P. 249

WILFRED LESLIE SLEIGHT, M.R.C.S., L.R.C.P. .. 250

Meetings of Societies .. .. .. .. .. .. .. 251

The Medical Library of the University of Bristol .. .. 254

Publications Received .. .. .. .. .. .. .. 257

Local Medical Notes .. .. .. .. .. .. .. 259

